# Pharmacists’ and patients’ perceptions about the importance of pharmacist services types to improve medication adherence among patients with diabetes in Indonesia

**DOI:** 10.1186/s12913-021-07242-1

**Published:** 2021-11-13

**Authors:** Bobby Presley, Wim Groot, Milena Pavlova

**Affiliations:** 1 Department of Health Services Research (HSR), Care and Public Health Research Institute (CAPHRI), Maastricht University Medical Center (MUMC+), Faculty of Health, Medicine and Life Sciences (FHML), Maastricht University, PO Box 616, Maastricht, MD 6200 The Netherlands; 2 Department of Clinical and Community Pharmacy, Center for Medicines Information and Pharmaceutical Care (CMIPC), Faculty of Pharmacy, University of Surabaya, Surabaya, East Java 60293 Indonesia

**Keywords:** Pharmacist, Diabetes, Medication adherence, Services

## Abstract

**Background:**

Various pharmacist services are available to improve medication adherence, including consultation, brochure, etc. Challenges arise on which services are best implemented in practice. Knowledge about patients’ and pharmacists’ preferences can help to prioritize services. This study explores the pharmacists’ and patients’ perceptions about the importance of pharmacist services to improve medication adherence among patients with diabetes in Indonesia.

**Methods:**

This questionnaire-based cross-sectional study involved adult outpatients with diabetes type 2 and pharmacists from community health centers (CHCs) and hospitals in Surabaya, Indonesia. Random sampling was used to identify 57 CHCs in the study. In addition, based on convenient sampling, three hospitals participated. All pharmacists working at the CHCs and hospitals, who were willing to participate, were included in the study. For patients, minimum sample size was calculated using Slovin’s formula.

Patients and pharmacists were asked to rank five pharmacist service types (consultation, brochure/leaflet, patient group discussion, medication review, and phone call refill reminder) according to their importance to improve medication adherence. A face validity test of the self-developed questionnaire was conducted before the data collection. Rank ordered probit models were estimated (STATA 15th software).

**Results:**

A total of 457 patients from CHCs, 579 patients from hospitals, and 99 pharmacists from both medical facilities were included. Consultation (CHC patients 56.0% vs hospital patients 39.7% vs pharmacists 75.2%) and brochure (CHC patients 23.2% vs hospital patients 27.5% vs pharmacists 11.9%) were the most preferred pharmacist services. Patients with experience getting medication information from pharmacists valued consultation higher than brochure and patient group discussions. Older patients ranked a brochure higher than other services. Patients without formal education in CHCs had a lower probability of giving a high rank to a brochure to improve medication adherence. There was significant positive correlation between the ranking of phone call refill reminder and medication review (0.6940) for patients in CHCs.

**Conclusion:**

For both patients and pharmacists, consultation, brochure, and group discussion were the highest-ranked services. Education, age, experience with pharmacist services, and medical facility features need to be considered when evaluating which pharmacist services to implement in Indonesia.

**Supplementary Information:**

The online version contains supplementary material available at 10.1186/s12913-021-07242-1.

## Introduction

Pharmacists play a role in patient care by providing medication information to patients, performing medication reviews, and monitoring medication use. These roles make it possible for pharmacists to collaborate with other healthcare professionals to optimize patients’ health outcomes, especially among patients with chronic diseases who continuously use medication. Medication adherence is essential to reduce the negative long-term consequences of chronic diseases. While physicians provide comprehensive care, pharmacists can support physicians by advising patients on their medication use and adherence [1]. The American Diabetes Association guideline recommends pharmacist’s involvement in patient care collaborating with other healthcare professionals [2]. This extended role of the pharmacist in diabetes care has been well documented in previous studies [3–7]. Even though evidence supports a greater role of the pharmacist, the possibility to integrate this role in practice needs to be carefully considered by exploring patients’ and pharmacists’ perspectives, especially in limited resources countries.

The pharmacist’s role in providing care to patients with diabetes focuses on appropriate, effective, and safe medication use [8–11]. Pharmacists can also be patient educators, refer patients to the physician in case of a disease or medication-related problem, and monitor patient medication use [1, 12–15]. Three basic approaches can be distinguished to accommodate these roles, namely educational, behavioral, and a combination of both approaches. These approaches have as a goal to change behavior, in particular, to improve medication adherence. Educational-based services focus on improving the patient’s disease and treatment management knowledge to become more aware of the condition. Behavioral-based services aim to enhance behavior [16, 17]. Combining these approaches can cover both areas and provide better outcomes in modifying behavior than the separate approaches [16–18]. Pharmacists commonly use these approaches to enhance medication adherence [16, 17]. Improvement of medication adherence can result in better health outcomes, particularly in well-maintained blood glucose target control [2]. Medication adherence can be enhanced in various ways, including education sessions, consultation, telephone calls, patient group discussions, printed or digital material. These services have been shown to improve medication adherence in patients with diabetes [3].

This study investigates the perspective of pharmacists and patients with diabetes in Indonesia regarding services to improve medication adherence. The prevalence of diabetes in Indonesia is high and increasing, according to the Indonesian National Report in 2018 [19]. Indonesia ranks 7th in the world based on the number of diabetes cases according to International Diabetes Federation (IDF) 2019 [20]. A study in three districts in Surabaya, Indonesia, also shows the high prevalence of medication non-adherence among patients with diabetes (81%) [21], which underlines the need of our study.

In Indonesia’s health system, which aims at universal health coverage, it is important to have a balanced ratio between healthcare professionals and patients to deliver optimal health services. In practice, the insufficient number of physicians in medical facilities in Indonesia, such as community health centers (CHCs) and hospitals, hamper the quality of health services [22]. Within these limitations, pharmacists in these facilities play an important role in supporting physicians in patient care through medication information, medication review, and medication monitoring [23, 24]. Based on these extended roles, pharmacists can help improve patient outcomes in collaboration with physicians. This study is relevant since the pharmacist’s role in Indonesia is still developing to focus more on patient care. The pharmacist is a relatively new health profession compared to other more established health professionals in Indonesia. Its role in patient care is not yet well recognized by other healthcare professionals, such as physicians, but also not by patients. Therefore, input from pharmacists and patients as the end-user can play an important role to identify suitable services to improve medication adherence. The pharmacists’ expanded role is regulated by the Indonesian Ministry of Health and the Indonesian Pharmacist Association through pharmaceutical care guidelines in CHCs and hospitals [23, 24]. However, not all pharmacists in Indonesia can implement the new extended role smoothly. Time constraints, the burden of administration, and a limited number of healthcare professionals, including pharmacists, hamper pharmacists’ involvement in patient care [25, 26]. Medicine information provision focusing on medication administration is the most common type of consultation pharmacists provide in practice. Hitherto, there is no consensus on the most suitable pharmacist services in CHCs and hospitals in Indonesia. Empirical evidence on the views of patients and pharmacists regarding the desirability of such services is lacking.

In view of this, it is necessary to explore patients’ and pharmacists’ perspectives to provide insight into preferred pharmacist services. Therefore, this study aims to identify the importance that patients and pharmacists attach to different pharmacist services to improve medication adherence among patients with diabetes (i.e., service ranking). We allow for differences in how patients (service users) and pharmacists (service providers) rank the pharmacist services. Therefore, combining evidence on the ranking of service importance by both patients and pharmacists can provide insight into the type of pharmacist services that should be offered to patients in Indonesia to improve their medication adherence.

## Methods

### Study design

This is an explanatory quantitative study with a cross-sectional design. A self-developed questionnaire was used for this survey. The data were questionnaire-based and were collected as part of a larger survey among patients with diabetes and pharmacists regarding their preferences for receiving/providing services to increase medication adherence. Here, we only analyzed the data on the ranking of the importance of selected pharmacist services by patients and pharmacists. The questionnaire was developed in English and then translated into the Indonesian language. The translated questionnaire was pre-tested among potential respondents to check the face validity of the questions and to improve the wording. Among others, the questionnaire included questions on the respondent’s preferences for five pharmacist services to improve medication adherence, namely face-to-face individual consultation, brochure/leaflet, patient group discussion (a scheduled meeting with several patients with diabetes to discuss and share information), medication review (pharmacist’s review of patient’s medication each time the patient has a medication refilled to prevent and manage any medication-related problems), and a phone call refill reminder. These pharmacist services were selected based on a prior systematic literature review to improve medication adherence among patients with diabetes [3]. Respondents were presented with five pharmacist services that were most frequently used according to the review. An explanation of each service was provided to the respondents. They were asked to rank the services (from 1 to 5, without repeating the number) based on the importance they attach to such services. Respondents were also asked which service they had already provided/received to improve medication adherence. The English wording of the questions used in this paper and details on the services are presented in Additional file 1.

### Ethical and access approval for data collection

Ethical approval was obtained from the Institutional Ethical Committee of the University of Surabaya (067/KE/II/2019). Approval for data collection in CHCs was obtained from the Surabaya City Health Office (072/9061/436.7.2/2019). Approval for data collection in hospitals was acquired by a letter from one public hospital (070/6236/43686/2019) and two private hospitals (Kp.2.07/2/18/PT.PHC-2019 and 934/RSHU/Dir./V/2019). Each participant was informed about the study and signed a written informed consent letter prior to the survey.

### Respondents and data collection

The data collection was carried out by the main author and a research assistant team (4 members). The data collection process took place in February–November 2019 in Surabaya, Indonesia.

Since implementing the universal health coverage program in Indonesia in 2014, the CHCs have become the first gatekeeper for the patient to get healthcare services, including patients with chronic diseases. Patients can also go to the hospital to get health services from the community health center. Patients can have medical care and medication prescription from the physician at those health facilities periodically. Patients cannot get their medication in community pharmacies without a prescription. Therefore, patients who go to CHCs and hospitals get the prescription directly after visiting the physician and receive their medication from the pharmacies in the facilities. Patients can get their medicines from community pharmacies if these are not available in the CHCs and hospitals. This study focuses on both CHCs and hospitals.

Thus, three groups of respondents were involved:

- The first group was pharmacists. Data collection was conducted in 57 CHCs and three hospitals (one public hospital and two private hospitals) in Surabaya, Indonesia.
- The second group was patients in 57 CHCs in Surabaya.
- Finally, we surveyed patients in outpatient clinics in three hospitals (one public hospital and two private hospitals) in Surabaya.

The main author or one of the research assistants conducted the interviews and explained the study to the respondents. Each respondent had to complete the questionnaire in the presence of the main author and/or research assistant.

### Sample size calculation

- Respondents in the CHCs

CHCs in this study covered the north, east, west, south, and center of Surabaya, Indonesia. Sample size calculation for the number of CHCs to be involved in each area for the collection of data among patients (second group of respondents) was done using Slovin’s formula:

$$ \mathrm{n}=\mathrm{N}/\left(1+\mathrm{N}\ {\mathrm{e}}^2\right) $$

Where N is the expected population size, and e is the error tolerance (0.05).

The minimum number of CHCs generated by the formula was 12 in the north, 14 in the south, 10 in the west, 13 in the east, and 8 in the center area. In the next step, we selected the community health center to be included in the study, based on the generation of random numbers using MS Excel on the list of all CHCs in each area.

In the data collection among patients in CHCs, a minimum target sample size of 391 respondents (for all 57 CHCs mentioned above) was calculated based on Slovin’s formula. Additional patients were sampled to account for the probability of dropout. Thus, the sample included 457 respondents from 57 CHCs (each community health center provided eight respondents).

- Respondents in hospitals

Only three hospitals were involved in the study because only those were willing to participate in the study. Data collection among patients in the hospital used the same formula for the sample size calculation with a minimum target sample size of 381 respondents. Again, patients were added to this minimum, and a total of 579 respondents were surveyed from the three hospitals (272 respondents for one public hospital and 307 respondents distributed in two private hospitals).

- Pharmacists as respondents in the community health center and hospital

To collect data among pharmacists, all pharmacists in the 57 CHCs and the three hospitals, who were willing to participate and had experience in providing services to outpatient with diabetes, were included in the study.

### Inclusion and exclusion criteria

All patients involved in this study were adult outpatients (≥ 18 years old) with diabetes type 2 who were taking diabetes medication and were visiting CHCs or hospitals for medication refills and/or routine control by the physician. Identification of patients with type 2 diabetes was made with the help of pharmacists in each medical facility. Respondents who had difficulty communicating with others and all inpatients with diabetes were excluded from the study. All pharmacists who worked in the CHCs and hospitals involved in the study were included. Pharmacists who refused to participate in the study were excluded.

### Data analysis

Descriptive analysis was used to analyze the socio-demographic data of the respondents (pharmacists and patients). This study applied a rank-ordered probit model to identify the influence of respondents’ characteristics on the pharmacist service ranking and identify the correlation between pharmacist services’ rankings conditional on the impact of observable traits. This analysis was done for both pharmacists and patients. This enabled to study differences in pharmacist service ranking between pharmacists and patients, especially in CHCs and hospitals. Rank ordered probit (ROP) models were estimated using maximum simulated likelihood (MSL) methods for pharmacists and patients. The models were used to analyze the relationship between the rank of the services assigned by the respondents and the respondents’ socio-demographic characteristics.

The ROP could be seen as an extension of an ordered probit model. This model for ranking data assumes a normal distribution of the error term [27]. It extends the descriptive statistics of the average ranking by respondents by the impact that observable characteristics have on this ranking. An additional advantage of the ROP model is that it accounts for the correlation of the error terms, conditional on the impact of the observed characteristics that influence the respondents’ ranking of alternatives [28]. By this, the ROP model also considers the dependencies of choices between rank levels. This results in more robust estimation and reduces misspecification of the error terms [27, 28]. Thus, our analysis provided insight into the importance of pharmacist services for respondents with specific socio-demographic characteristics to improve medication adherence. We estimated two models: the first model only included the services (intercepts), and the second model had an additional set of explanatory variables (socio-demographic characteristics). These two models were estimated for pharmacists and patients separately.

The marginal effect of each respondents’ characteristic on the ranking of a given service was also calculated. The correlation matrix showed how the ranking of one service was associated with the ranking of another service. The marginal effects were calculated by multiplying the marginal effect of a given characteristic with the overall probability of each type of service being ranked as most preferred. This was added to the overall probability of each service. The sign of the marginal effect of a given socio-demographic characteristic for a given service indicated the direction in which the probability of that service being ranked as the most preferred changes for a one-unit change in the given characteristic (keeping all other variables constant).

## Results

In total, 457 patients from CHCs, 579 patients from hospitals with a response rate of 91.95 and 95.07%, respectively, and 99 pharmacists (response rate 100%) participated in this study. Details about patients’ characteristics are presented in Additional file 2. The results of independent t-tests, the Two-sample Wilcoxon rank-sum (Mann-Whitney) test, and the Kruskal-Wallis equality-of-populations rank test are included in Additional file 2 to compare the characteristics of the patients included in the study. The respondents from CHCs and hospitals were statistically different in all the socio-demographic characteristics, except for sex and marital status. Therefore, we conducted the analysis separately for patients in CHCs and hospitals. Patients in CHCs and hospitals had an average age of 59 and 61 years, respectively. Many patients with comorbidities were treated in a hospital (67.61% vs. 82.73%). Almost half of the patients in the hospitals (43.90%) and a quarter of patients in the CHCs (26.70%) had never received any of the five pharmacist service types. Consultation, brochure, and patient group discussion were the top three pharmacist services used by patients in the CHCs. In hospitals, the results were slightly different. In particular, brochure, consultation, and phone call refill reminders were the top three pharmacist services used by patients in hospitals.

Pharmacists in CHCs and hospitals involved were young, with an average age of 32.02 years, with only a small number of pharmacists with a master’s degree (7.1%). Consultation and brochure were the two most frequently used services by pharmacists to help patients with their medication. In general, pharmacists in both medical facilities had already used brochures, consultation, and patient group discussion before. Details about the pharmacist’s characteristics in this study are presented in Additional file 3.

### Ranking of service by patients in CHCs and hospitals

Patients in this study were provided with details on the pharmacist services that can improve medication adherence and were asked to rank these services. Patients in CHCs were more frequently assigned rank 1 (most important) to pharmacist consultation (56%) and brochure (23.20%) compared with the other services. A similar result was found in hospitals for consultation (39.70%) and brochure (27.50%). This higher rank indicated preferences for these services to enhance medication adherence. Patient group discussion, medication review, and phone call refill reminders were less preferred services in both medical facilities. Details on the ranking can be found in Table 1.

**Table 1:** Pharmacist service ranking according to patients with diabetes in community health centers (CHCs) and hospitals

Ranking	Pharmacist services									
	Brochure/leaflet		Face to face individual consultation		Patient group discussion		Medication review		Phone call refill reminder	
	CHCs	Hospitals	CHCs	Hospitals	CHCs	Hospitals	CHCs	Hospitals	CHCs	Hospitals
1	106 (23.20%)	159 (27.50%)	256 (56.00%)	230 (39.70%)	31 (6.80%)	38 (6.60%)	40 (8.80%)	68 (11.70%)	24 (5.30%)	84 (14.50%)
2	84 (18.40%)	82 (14.20%)	125 (27.40%)	215 (37.10%)	167 (36.50%)	80 (13.80%)	58 (12.70%)	132 (22.80%)	23 (5.00%)	70 (12.10%)
3	110 (24.10%)	108 (18.70%)	52 (11.40%)	78 (13.50%)	172 (37.60%)	217 (37.50%)	101 (22.10%)	108 (18.70%)	22 (4.80%)	68 (11.70%)
4	80 (17.50%)	116 (20.00%)	17 (3.70%)	43 (7.40%)	64 (14.00%)	128 (22.10%)	240 (52.50%)	217 (37.50%)	56 (12.30%)	75 (13.00%)
5	77 (16.80%)	114 (19.70%)	7 (1.50%)	13 (2.20%)	23 (5.00%)	116 (20.00%)	18 (3.90%)	54 (9.30%)	332 (72.60%)	282 (48.70%)
Mean ± standard deviation	2.86 ± 1.395	2.90 ± 1.491	1.67 ± 0.925	1.95 ± 1.015	2.74 ± 0.955	3.35 ± 1.141	3.30 ± 1.035	3.10 ± 1.199	4.42 ± 1.129	3.69 ± 1.518
Median	3.00	3.00	1.00	2.00	3.00	3.00	4.00	3.00	5.00	4.00

### Ranking of services by pharmacists in CHCs and hospitals

Pharmacists were also asked to rank the five pharmacist services according to their preference to provide them to improve medication adherence among patients with diabetes. Consultation and brochures were the most preferred pharmacist services. This result was similar to the results above among patients. Details on pharmacist ranking are presented in Table 2.

**Table 2:** Pharmacist service ranking according to pharmacists

Pharmacist service	Ranking					Mode	Mean	Median	Standard deviation
	1	2	3	4	5				
Brochure/leaflet	12 (11.90%)	32 (31.70%)	23 (22.80%)	14 (13.90%)	18 (17.80%)	2	2.94	3.00	1.300
Face to face individual consultation	76 (75.20%)	14 (13.90%)	6 (5.90%)	2 (2.00%)	1 (1.00%)	1	1.36	1.00	0.775
Patient group discussion	3 (3.00%)	31 (30.70%)	24 (23.80%)	24 (23.80%)	17 (16.80%)	2	3.21	3.00	1.154
Medication review	3 (3.00%)	13 (12.90%)	30 (29.70%)	38 (37.60%)	15 (14.90%)	4	3.49	4.00	1.004
Phone call refill reminder	5 (5.00%)	9 (8.90%)	16 (15.80%)	21 (20.80%)	48 (47.50%)	5	3.99	5.00	1.216

### Rank ordered probit model analysis for types of pharmacist service (patients and pharmacists)

Table 3 presents the result of the first rank-ordered models for patients and pharmacists. As indicated by the table, most respondents gave lower rank to other pharmacist services (brochure, patient group discussion, medication review, and phone call refill reminder) than consultation. Differences in the rank order between patients and pharmacists were observed, especially for services ranked as second/third important after consultation (Table 3). A phone call refill reminder was the least preferred type of service in all respondent groups. These results were similar to the rank order above (Tables 1 and 2). The regression coefficients in Table 3 provide information on the differences in the relative importance of pharmacist services from the respondents’ perspective.

**Table 3:** The first rank-ordered probit model for the type of pharmacist services (patients and pharmacists)

Type of service	Community health center patients		Hospital patients		Pharmacists	
	Coefficient	Standard error	Coefficient	Standard error	Coefficient	Standard error
**Consultation**	Reference					
**Brochure/leaflet Constant**	− 0.8375*	0.0849	− 0.5637*	0.0718	−1.3265*	0.1998
**Patient group discussion Constant**	− 0.7280*	0.0624	− 0.9011*	0.0637	− 1.4432*	0.1849
**Medication review Constant**	− 1.2968*	0.0983	− 0.7950*	0.0621	−1.6164*	0.1960
**Phone call refill reminder Constant**	−2.8950*	0.2341	−1.5036*	0.1164	−2.0216*	0.2517
**The covariates of patient group discussion**	−0.3240*	0.0760	−0.1208*	0.0612	−0.2976	0.1873
**The covariates of medication review**	0.0532	0.0834	−0.1143	0.0668	−0.4321*	0.1863
**The covariates of phone call refill reminder**	0.3762*	0.1091	0.4765*	0.0753	0.0089	0.1995
**/12_1**	0.3498*	0.0613	0.5412*	0.0610	0.6466*	0.1491
**/13_1**	0.2687*	0.0910	0.1028	0.0655	0.7612*	0.1348
**/14_1**	−0.0096*	0.1617	−0.2368*	0.1115	0.7199*	0.1853
**/13_2**	0.5777*	0.0889	0.4734*	0.0659	0.2228	0.1426
**/14_2**	0.6469*	0.1573	0.3683*	0.1090	0.1255	0.2012
**/14_3**	1.3378*	0.1612	0.6073*	0.1081	0.3666*	0.1761
**Observations (respondents)**	457		579		99	
**Wald chi2**	–		–		–	
**Log simulated-likelihood**	− 1583.4162		− 2402.7042		− 365.19124	

*P < 0.05

### Rank-ordered probit model including explanatory variables - patients

As shown in Table 4, the ranking of services by patients in CHCs depended on observable characteristics, such as the source of medication information, total monthly income, educational background, comorbidities, and the ability to cover household expenses. The interpretation of Table 4 is based on the regression coefficients and the constant terms of each pharmacist service. A positive or negative sign of the regression coefficient indicates that a characteristic increases or decreases the rank order of a certain type of pharmacist service compared with consultation. Overall, consultation was the most preferred service to improve medication adherence based on the estimations’ constant terms. Following consultation, the rank order was brochure, patient group discussion, medication review, and phone call refill reminder. Patients who had experience getting medication information from pharmacists valued consultation significantly higher than the other services (see regression coefficients in Table 4). Similar results were found among patients with a total monthly income of 96 USD (1.400.000 IDR) or higher. Patients with a higher income gave lower ranking to brochure, group discussion, and medication review than a consultation to improve medication adherence. Older respondents who visit CHCs were more likely to rank brochure higher than consultation. Table 4 also shows that the source of medication information was a patient characteristic that contributed to the ranking of pharmacist services regarding hospital patients. The same held for the ability to cover household expenses and educational background. Patients who had experience getting medication information from pharmacists, tended to rank brochure and patient group discussion lower than consultation. At the same time, a phone call refill reminder was more likely to be ranked higher compared to consultation based on the regression coefficient and the constant term in Table 4, especially for patients who had experience getting medication information from pharmacists. Further results can be found in Table 4.

**Table 4:** Final model on the rank-ordered probit model analysis (patients)

Type of service	Community health centers		Hospitals	
	Coefficient	Standard error	Coefficient	Standard error
**Consultation (references)**				
**Brochure/leaflet**				
Age	0.0251*	0.0104	0.0054	0.0090
Female**	0.2587	0.2191	0.1581	0.1739
Married**	0.0198	0.2012	0.1070	0.1689
No formal education 1**	−1.0092*	0.4704	0.6333	0.4456
Primary education background**	−0.0662	0.3353	− 0.2148	0.2529
Secondary education background**	− 0.1415	0.3140	− 0.0060	0.2234
Work**	0.1838	0.2004	0.0433	0.1955
Inability to cover household expenses**	−0.5487*	0.2333	0.3019	0.2089
Needs help to take medication**	−0.6087	0.3633	−0.2648	0.1789
Have experience of missing to take medication**	−0.2030	0.1703	0.0006	0.1534
With comorbidities**	0.0558	0.1793	−0.0741	0.1846
Experiences to get medication information from pharmacist**	−1.7560*	0.1916	−0.7225*	0.1406
Monthly income1**	−0.7888*	0.1857		
Monthly income2**			0.2173	0.1739
Constant	−0.8191	0.8233	−0.7096	0.7202
**Patient group discussion**				
Age	0.0105	0.0081	0.0036	0.0072
Female**	0.1360	0.1581	0.2636	0.1380
Married**	0.0507	0.1434	0.0843	0.1329
No formal education 1**	−0.0022	0.3470	0.3179	0.3455
Primary education background**	0.4484	0.2500	0.0211	0.2018
Secondary education background**	0.3150	0.2370	0.1450	0.1795
Work**	−0.0012	0.1481	− 0.0057	0.1537
Inability to cover household expenses**	0.0148	0.1609	0.3483*	0.1598
Needs help to take medication**	−0.1293	0.2525	0.0295	0.1395
Have experience of missing to take medication**	−0.0680	0.1221	0.0510	0.1211
With comorbidities**	0.2638*	0.1300	−0.2428	0.1435
Experiences to get medication information from pharmacist**	−0.4270*	0.1320	−0.2624*	0.1102
Monthly income1**	−0.2750*	0.1318	0.0876	0.1376
Monthly income2**			−1.2646*	0.5756
Constant	−1.6872*	0.6643		
**Medication review**				
Age	0.0047	0.0111	−0.0012	0.0070
Female**	0.0450	0.2214	0.0977	0.1369
Married**	0.0282	0.2006	0.0553	0.1314
No formal education 1**	−0.2791	0.4805	−0.7683*	0.3853
Primary education background**	0.8882*	0.3480	−0.0230	0.1993
Secondary education background**	0.3950	0.3274	0.0711	0.1770
Work**	−0.1343	0.2075	−0.2045	0.1557
Inability to cover household expenses**	0.8531*	0.2260	0.0946	0.1631
Needs help to take medication**	0.0131	0.3445	0.1540	0.1386
Have experience of missing to take medication**	−0.2197	0.1727	0.0123	0.1201
With comorbidities**	0.1898	0.1797	−0.0780	0.1440
Experiences to get medication information from pharmacist**	−0.7446*	0.1875	0.0033	0.1086
Monthly income1**	−0.5820*	0.1861		
Monthly income2**			−0.1148	0.1374
Constant	−1.7710*	0.9030	−0.7722	0.5680
**Phone refill reminder**				
Age	0.0032	0.0180	0.0102	0.0120
Female**	−0.0301	0.3781	0.0783	0.2314
Married**	0.0507	0.3501	−0.3810	0.2238
No formal education 1**	−0.8877	0.8696	−1.1793	0.6712
Primary education background**	1.4623*	0.6292	0.1348	0.3315
Secondary education background**	0.5219	0.5896	0.0540	0.2910
Work**	0.0959	0.3541	0.2217	0.2634
Inability to cover household expenses**	1.6594*	0.4042	−0.3739	0.2933
Needs help to take medication**	0.1927	0.6034	0.0338	0.2384
Have experience of missing to take medication**	−0.2610	0.3038	0.3432	0.2030
With comorbidities**	0.3232	0.3182	0.2730	0.2530
Experiences to get medication information from pharmacist**	0.3101	0.3702	0.4583*	0.1876
Monthly income1**	−0.6019	0.3258		
Monthly income2**			0.3809	0.2306
Constant	−4.8354*	1.4634	−2.6908*	0.9730
The covariates of patient group discussion	−0.1399	0.0790	− 0.0916	0.0622
The covariates of medication review	0.1691*	0.0853	−0.0880	0.0675
The covariates of phone call refill reminder	0.5145*	0.1099	0.4964*	0.0762
/12_1	0.3405*	0.0744	0.5355*	0.0632
/13_1	0.1364	0.1105	0.1177	0.0674
/14_1	−0.0105	0.1896	−0.1794	0.1138
/13_2	0.6921*	0.1061	0.4751*	0.0682
/14_2	0.6890*	0.1964	0.4136*	0.1129
/14_3	1.4939*	0.1864	0.6194*	0.1123
Observations (respondents)	457		577	
Wald chi2	164.48*		83.82*	
Log simulated likelihood	− 1471.7322		− 2342.8133	

*P < 0.05;** Sex: 1 = female; Marital status: 1 = married/living together; Education 1: 1 = no formal education; Education 2: 1 = primary education; Education 3: 1 = secondary education; Work status: 1 = work; Household: 1 = total income cannot cover household expenses; Needs help: 1 = need help to take medication; Missed medication: 1 = have experience missing to take medication; Comorbidities: 1 = have the comorbid disease; Experiences to get medication information from pharmacist: 1 = pharmacist; Monthly income1: 0 = < 96 USD (1.400.000 IDR), 1 = ≥ 96 USD (1.400.000 IDR); Monthly income2: 0 = < 138 USD (2.000.000 IDR), 1 = ≥ 138 USD (2.000.000 IDR)

As shown in Table 5, there was a significant positive correlation between phone call refill reminders and medication review (0.6940; *p* < 0.05) for patients in CHCs. This correlation meant that patients in CHCs who gave a higher or lower rank to phone call refill reminders were more likely to rank medication reviews higher or lower, respectively. On the other hand, there was a significant correlation between patient group discussion and brochure (0.5061, *p <* 0.05) for hospital patients. This positive correlation meant that given the observable characteristics, patients in hospitals who ranked group discussion higher also ranked brochure higher.

**Table 5:** Rank correlation final model with consultation as reference (Patients and pharmacists)

**Patients in community health centers**				
	Brochure/leaflet	Patient group discussion	Medication review	Phone call refill reminder
Brochure/leaflet	1.0000			
Patient group discussion	0.3646*	1.0000		
Medication review	0.0989	0.5036*	1.0000	
Phone call refill reminder	−0.0045	0.2718*	0.6940*	1.0000
**Patients in hospitals**				
	Brochure/leaflet	Patient group discussion	Medication review	Phone call refill reminder
Brochure/leaflet	1.0000			
Patient group discussion	0.5061*	1.0000		
Medication review	0.1133	0.4520*	1.0000	
Phone call refill reminder	−0.0990	0.1467*	0.3946*	1.0000
**Pharmacists**				
	Brochure/leaflet	Patient group discussion	Medication review	Phone call refill reminder
Brochure/leaflet	1.0000			
Patient group discussion	0.6067*	1.0000		
Medication review	0.7150*	0.6325	1.0000	
Phone call refill reminder	0.5366*	0.4459	0.5403	1.0000

*P < 0.05

### Rank-ordered probit model including explanatory variables - pharmacists

Table 6 presents the rank-ordered model for pharmacists after including observable pharmacists’ characteristics. However, this model was not statistically significant (Wald chi-square: 20.82, *p* > 0.05). The results meant that no significant variation was explained by the pharmacists’ variables included in the model. The correlation matrix in Table 6 shows a strong correlation between the pharmacists’ ranking of brochure and the ranking they gave to other types of pharmacist services.

**Table 6:** Final model on the rank-ordered probit model analysis (pharmacists)

Type of service	Coefficient	Standard error
**Consultation references**		
**Brochure/leaflet**		
Age	0.0448	0.0563
Female	−0.3980	0.5056
Master degree	−0.3331	0.8339
Have experience helping non-adherence patient	−0.7697*	0.3874
Work duration of ≥7.75 years	−0.0541	0.6255
**Constant**	−2.0314	1.6168
**Patient group discussion**		
Age	0.0078	0.0464
Female	−0.6881	0.4008
Master degree	−0.6653	0.8087
Have experience helping non-adherence patient	−0.3227	0.3175
Work duration of ≥7.75 years	0.2605	0.5170
**Constant**	−1.1068	1.3352
**Medication review**		
Age	−0.0188	0.0478
Female	−0.2263	0.4262
Master degree	−0.7114	0.8180
Have experience helping non-adherence patient	−0.7663*	0.3330
Work duration of ≥7.75 years	0.2651	0.5324
**Constant**	−0.6080	1.3569
**Phone refill reminder**		
Age	−0.0084	0.0578
Female	0.2747	0.5436
Master degree	−0.9476	0.9155
Have experience helping non-adherence patient	−0.6113	0.3810
Work duration of ≥7.75 years	−0.5121	0.6531
**Constant**	−1.4649	1.6440
**The covariates of patient group discussion**	−0.2690	0.1929
**The covariates of medication review**	−0.4546*	0.1877
**The covariates of phone call refill reminder**	0.0248	0.1979
/12_1	0.5832*	0.1577
/13_1	0.6950*	0.1408
/14_1	0.6792*	0.1931
/13_2	0.2430	0.1473
/14_2	0.1915	0.2061
/14_3	0.2304	0.1920
Observations (respondents)	99	
Wald chi2	20.82	
Log simulated likelihood	−348.86853	

*P < 0.05

### Marginal effect analysis of patients with diabetes

Additional file 4 presents the marginal effects of patients’ characteristics on the ranking of pharmacist services. Patients who had experience getting medication information from pharmacists had a significantly lower probability of choosing brochures as the preferred service in both CHCs (decrease 6.72% points) and hospitals (decrease 4.54% points). The likelihood of brochures to be ranked as number one was also found to be lower for patients without formal education and patients with a total monthly income of 96 USD (1.400.000 IDR) or higher in CHCs. At the same time, patients with experience of getting medication information from the pharmacist in both medical facilities had a significantly higher probability of being chosen as the preferred pharmacist service to improve their medication adherence. Similar results were also found among patients in CHCs, who had a total monthly income ≥ of 96 USD (1.400.000 IDR) or higher. Details on the marginal effect can be found in Additional file 4.

### Marginal effect analysis of pharmacists

The marginal effects for the pharmacist ranking are presented in Additional file 5. Overall, the marginal effects based on the pharmacist data show non-significant results even though some have a *P*-value close to < 0.05.

## Discussion

In general, consultation and brochure were the two most preferred types of pharmacist services by patients and pharmacists. These results were similar to the first rank model analysis. Consultation was considered the most preferred type of pharmacist service by all respondent groups. This finding aligns with previous systematic literature in which consultation was one of the most common types of pharmacist services used to improve medication adherence [3]. Patients’ high rank for consultation might be influenced by the need for services that provide the possibility for face-to-face interaction between pharmacists and patients to discuss and solve medication-related problems [29–31]. This finding is in line with other studies, which show patients’ expectation of having a pharmacist who takes the role of an educator through consultation/counseling about the disease or medication, especially if physicians are too busy to provide information and answer patients’ queries [32, 33]. From a pharmacists’ point of view, consultation is an important part of pharmaceutical care services [34]. If consultation is reimbursed separately from handing out medication, it is an additional source of income for pharmacists. As one of the more accessible healthcare providers in the community, pharmacists can provide necessary medication-related information. The pharmacists’ position can have potential benefits, especially due to the possibility of regular contact with patients with chronic diseases. In particular, pharmacists can identify and monitor medication-related problems, including medication adherence [29, 31]. This is supported by the standard of pharmaceutical care in Indonesia, which stipulates that consultation is part of pharmacist services that should be given to the patient to improve medication adherence [23, 24]. Through consultation, pharmacists can identify and recommend solutions for medication-related problems. Patients might also think that discussing their medication problems with pharmacists is easier than with physicians. Most patients are comfortable discussing their diabetes and medication with their pharmacists compared with other health care professionals [35]. Professional relationships between pharmacists and patients can be built through consultation to support the pharmacist’s role in patient care, including medication adherence monitoring [15]. This finding shows the important role of consultation for both patient and pharmacist as the most suitable service to help improve medication adherence. The Indonesian Pharmacist Association and pharmacists need to work together to evaluate current practice and identify limitations in pharmacists’ practice that can hinder the provision of patient care-based services, including consultation [25, 26].

Brochure was the other favored pharmacist service by patients and pharmacists to improve medication adherence. A brochure might be seen as an easy and low-cost way to deliver information. Several reasons might be behind these findings in the Indonesian context, such as the high number of patients who visit medical facilities, time constraints, and a lack of pharmacists [25, 26].

As shown in the full model and the marginal effects, patients in both medical facilities who had experience getting medication information from the pharmacist valued consultation higher than a brochure and patient group discussion. While patients in CHCs valued medication review lower than consultation. This finding is similar to the finding of another Indonesian study reporting that patients who have experience getting medication information from pharmacists do not find it important to have a medication review added to the consultation [36]. Their experiences and potential benefits from pharmacist consultation during their visit to CHCs might explain these findings, even though further study is needed to confirm this hypothetical explanation. Differences in experience with pharmacist services can result in different expectations, i.e., more experienced patients may expect pharmacists to provide medication information spontaneously instead of waiting for patients to ask for it [37].

Total monthly income seemed not to influence patients in hospitals regarding their ranking of pharmacist services. On the other hand, total monthly income influenced patients’ ranking for pharmacist services, especially patients in CHCs. The reason behind these differences was not entirely apparent. The composition of patients in hospitals and CHCs may also explain these differences. Most hospital patients did not have much experience with pharmacist services, including consultation, compared with patients in CHCs (Additional file 2). Lack of exposure to pharmacist services may influence the ranking of pharmacist services to improve medication adherence among hospital patients. The differences in health conditions between both groups of patients, CHCs patients and hospital patients, might also influence the difference in the ranking of pharmacist services.

At the same time, older patients in CHCs ranked a brochure higher compared to consultation. This result was also reflected in the marginal effects showing that older patients had a higher probability of choosing a brochure. Obtaining support and information through a brochure is less time-consuming and more practical than consultation. Patients might need to wait for the consultation, which could be more uncomfortable for older people [38, 39], because sometimes getting the medication requires a long waiting time in medical facilities [38–41]. The long waiting time for patients to get their medication needs to be carefully handled to optimize pharmacist services in patient care.

Education influenced the ranking of the types of pharmacist services. Patients had a tendency not to choose services that look complicated such as medication review or brochure (Additional file 4), where patients without formal education in CHCs had a lower probability of selecting a brochure to improve their medication adherence. Lack of (health) literacy is a possible reason for the lower ranking of brochures. Reading written information in brochures may be more difficult than verbal communication through consultation or group discussion. The latter may negatively impact patients’ health conditions, including self-care management and decrease adherence among chronic disease patients [42, 43]. Therefore, pharmacists must carefully identify and apply different approaches for providing suitable services for patients with low (health) literacy in Indonesia. On the other hand, patients without formal education who visit hospitals also have a lower probability of choosing medication reviews as the preferred services. The possible reason is the extra time needed to review all the medication, while patients already need to endure long waiting times to get medical services [38].

The non-significant results of the full model analysis and the marginal effect analysis for pharmacists are most likely due to a lack of statistical power due to a small number of observations, a more homogenous sample, or less variation in the ranking. However, the pharmacists’ perspective results are still useful as they give insight into their overall ranking of the types of services. Further study involving more pharmacists from different cities could help establish whether the lack of significant differences among the pharmacists we observed is due to the small sample size in our study or a genuine homogenous ranking.

This study showed the perspective of patients and pharmacists on the ranking of pharmacist services that can help improve medication adherence among patients with diabetes. Not all of the results can be compared with previous studies due to the lack of similar studies. Most of the published studies analyze the effectiveness of pharmacist services to improve medication adherence and diabetes treatment goals. Even though comparison with similar studies is limited, these findings show that pharmacist services with a high ranking are in line with patient expectations found in other studies [32, 33] and the effectiveness of the services in improving diabetes treatment goals and medication adherence [3–7].

The findings can help enrich the development and promotion of the pharmacists’ role in patient care, especially in diabetes care and medication adherence. Patients’ ranking of pharmacist services can give information on services that can be prioritized to evaluate and modify services based on patients’ needs and pharmacists’ perspectives to improve medication adherence. The Indonesian Pharmacist Association may gain insight from the findings of this study to facilitate pharmacists to improve their role in patient care, especially in diabetes care. The findings of this study can provide important information to negotiate and discuss with other healthcare professionals, especially physicians and which roles in patient care can be delegated to the pharmacist. Further study to discuss the extended role of pharmacists in patient care by involving medical facilities representatives, physicians, or the Indonesian Pharmacist Association is needed to design suitable pharmacist services and explore the feasibility for implementation in practice.

This study has some limitations that need to be acknowledged. First, the study results may be only applicable to CHCs with pharmacists because many CHCs in Indonesia do not have pharmacists. Second, the three hospitals involved in this study are linked to an educational institution. Therefore, it is unclear if the findings are generalizable to the hospitals in other cities, especially in rural areas that do not have an education institution. Third, we were unable to perform any reliability tests. However, the ranking approach to study the attractiveness of certain items (in this study, what kind of pharmacist services that respondent wants to have) has been widely applied in research and is shown to be more reliable than the alternative approach of rating each item separately [44]. Furthermore, this study could not determine the construct validity or content validity tests since no prior expectations about the outcomes as this is the first study in Indonesia. Fourth, this study used a ranking-based approach without repeating the rank number of pharmacist services. This approach has the advantage of forcing respondents to make a tradeoff between pharmacist services. However, there is a possibility that some participants would have liked to give the same rank on some pharmacist services. The literature suggests combining ranking and rating measures [45], which we have not done in our study but could be relevant for further research. Fifth, the pharmacist services involved in this study were the most frequently used services based on the published literature. Further research could also include other pharmacist services less frequently used for the improvement of medication adherence among patients with diabetes, as well as services not yet available in the published literature.

## Conclusion

In this study, consultation is the highest-ranked pharmacist service for patients and pharmacists in Indonesia to improve medication adherence among patients with diabetes. Brochure, medication review, and patient group discussion are the other services that can be provided as a single service or in combination with a consultation. Age, total monthly income, medical facilities where patients get their medical services, and experiences with pharmacists’ medication information provision need to be considered when choosing suitable pharmacist services that meet patients’ and pharmacists’ preferences. Further study to explore the reasons behind the ranking of pharmacist services is needed to provide more insight into the services and their implementation in practice, especially within the Indonesian context. Future studies could also use both ranking and rating measurements and a broader range of services to obtain complete information about pharmacists’ and patients’ perceptions about the importance of pharmacist services.

## Supplementary Information

**Additional file 1.**

**Additional file 2.**

**Additional file 3.**

**Additional file 4.**

**Additional file 5.**

## Data Availability

The datasets used and/or analyzed during the current study are available from the corresponding author on reasonable request.

## Abbreviations

CHC: Community Health Center
IDF: International Diabetes Federation
ROP: Rank Order Probit
MSL: Maximum Simulated Likelihood
USD: United States Dollar
IDR: Indonesian Rupiah
