# Supplementary material for: Pharmacists’ and patients’ perceptions about the importance of pharmacist services types to improve medication adherence among patients with diabetes in Indonesia
Source: BMC Health Serv Res. 2021 Nov 13;21:1227. doi: 10.1186/s12913-021-07242-1 (PMC8590236; doi:10.1186/s12913-021-07242-1)
Supplement: Supplementary file 1 — Additional file 1. [file 12913_2021_7242_MOESM1_ESM.docx]

**Additional file 1. English wording on the questionnaire for patient and pharmacist**

1. **Questionnaire for patients**

| ***Section A: health and diabetes status data*** *(please fill in the table provided below and circle for your answer regarding a choice question*) | |
| --- | --- |
| *Q1. For how many years do you have your diabetes?* | _____ years |
| *Q2. Diabetes medication that you take currently* | |
| *Number of medication* | Number of oral: ____  Number of injection: _____ |
| *Name of medication* | Oral:  1. ____________  2. ____________  3. ____________  4. ____________ |
|  | Injection:  1. ____________  2. ____________  3. ____________  4. ____________ |
| *Frequency of administration (per day)* | Oral:  1. ____________  2. ____________  3. ____________  4. ____________ |
|  | Injection:  1. ____________  2. ____________  3. ____________  4. ____________ |
| *Q3. Do you have any other disease?* | 1. No other diseases 2. Hypertension 3. Heart disease 4. Stroke 5. Dyslipidemia 6. Renal problem 7. Vision-reducing problem or vision loss problem 8. Diabetic foot ulcer 9. Others ___________ |
| *Q4. What medication other than diabetes medication do you take now?* | 1. Prescription: 2. Non-prescription: 3. None |
| *Q5. Do you need help from others to take your medication?(i.e. help you to administer your medication)* | 1. Yes 2. No |
| *Q6. How often do you visit your physician per year to consult your clinical condition related to diabetes?* | ______ times |
| *Q7. Have you* ***ever missed*** *taking your medication or* ***modify the regimen*** *provided by the healthcare professionals in the past two months?* | 1. Yes 2. No |
| *Q8. Who gave you information about your medication?* | 1. Physician 2. Nurse 3. Pharmacist 4. Others __________ |
| *Q9. Have you received information about your medication from your pharmacist?* | 1. Yes (**please go to Q10)** 2. No (**please go to section B)** |
| *Q10. From which pharmacist did you get your information about your medication?* | 1. Hospital pharmacy 2. Community pharmacy 3. Others __________ |
| *Q11. Do you satisfy with the overall pharmacist’ services to help you understanding your medication that you get until now?* | 1. Very satisfied 2. Satisfied 3. Dissatisfied 4. Very dissatisfied |

**Patient characteristic**

| ***Social demographic*** |  |
| --- | --- |
| *Sex* | 1. Male 2. Female |
| *Age* | ______ years old |
| *Marital status* | 1. Single 2. Married/living together 3. Widowed/divorced |
| *The highest attained formal educational background* | 1. No formal education 2. Elementary education 3. Junior high school 4. Senior high school 5. Diploma 6. Bachelor’s degree 7. Master’s degree 8. Doctoral degree |
| *Work status* | 1. Work 2. Retired 3. Do not work |
| *Monthly income* | 1. < 96 USD (1.400.000 IDR) 2. 96 USD (1.400.000 IDR) – 137 USD (1.999.000 IDR) 3. 138 USD (2.000.000 IDR) – 239 USD (3.499.000 IDR) 4. 240 USD (3.500.000 IDR) – 343 USD (5.000.000 IDR) 5. > 343 USD (5.000.000 IDR) |
| *Do you think that your income could cover your household expenses?* | 1. Yes 2. No |
| *Health insurance (you can choose more than one)* | 1. Government insurance (BPJS) 2. Private insurance 3. No insurance 4. Others __________ |

**Section B. Patients' preferences toward the type of pharmacist services**

Below are some types of services that might be offered by pharmacists to help you take your diabetes medication consistently as instructed or indicated in the prescription:

- **The pharmacist provides information** regarding the risk factor of getting diabetes complications, the intended blood glucose level and other measurements that may influence the development of complications (for instance, blood pressure and low-density lipoprotein/LDL) **through**:
  1. ***Brochures/leaflet***
  2. ***Face to face individual consultation with pharmacist***
  3. ***Patient group discussion*:** a scheduled meeting with other patients with diabetes to discuss and share information
- ***Medication review***: pharmacist will review your medication each time you have your medication refilled to prevent and manage any medication-related problems
- ***Phone call refill reminder***: pharmacist will remind you to visit your physician and refill your medication by calling you two or three days before your schedule

Please rank the five pharmacist services that you really want to have by placing them in order of preference (1= the most preferred pharmacist service; and 5= the least preferred pharmacist service). Please only use **ONE number for ONE service**. Please also identify the type of pharmacist services that you have already experienced until now by giving mark (V) in the box.

| **Pharmacist services** | **Fill in the rank from 1-5.** | **Please** mark (V)  **to the type of services that you have you already experienced until now** |
| --- | --- | --- |
| Brochure/leaflet |  |  |
| Face to face individual consultation with the pharmacist |  |  |
| Patient group discussion |  |  |
| Medication review |  |  |
| Phone call refill reminder |  |  |

1. **Questionnaire for pharmacists**

***Section A: pharmacist characteristics*** *(please fill in the table provided below and circle your answer regarding choices based questions*)

| *Q1. Sex* | 1. Male 2. Female |
| --- | --- |
| *Q2. Age* | _____ years old |
| *Q3. The highest attained formal educational background* | 1. Apothecary 2. Master’s degree 3. Doctoral degree |
| *Q4. How long have you worked as a pharmacist?* | _____ years |
| *Q5. Which medical institution do you belong to?* | 1. Hospital 2. Primary health care center (Puskesmas) 3. Other _________ |
| *Q6. Do you give any instruction/information on how should* ***newly diagnosed diabetes patients*** *take their medication for the first time?* | 1. Yes **(please go to Q7)** 2. No **(please go to Q8)** |
| *Q7. What kind of information/instruction do you provide to* ***newly diagnosed diabetes patients*** *when they are taking their medication?* | 1. _______________ 2. _______________ 3. _______________ 4. _______________ 5. _______________ |
| *Q8. Do you give any instruction/information on how should* ***the regular patients with diabetes*** *take their medication?* | 1. Yes **(please go to Q9)** 2. No **(please go to Q10)** |
| *Q9. What kind of information/instruction do you provide to* ***the regular patient with diabetes*** *when they are taking their medication?* | 1. _______________ 2. _______________ 3. _______________ 4. _______________ 5. _______________ |
| *Q10. Do you give information on the importance of medication adherence to your patient with diabetes?* | 1. Yes **(please go to Q11)** 2. No |
| *Q11. How often do you ask your patients with diabetes about their medication adherence?* | 1. Once 2. Few times 3. Always 4. Never 5. others |
| *Q12. How many patient with diabetes did you have in* ***the past one months****?* | ______ patients |
| *Q13. What* ***type of services*** *and* ***how often*** *do you usually provide the services to your patients with diabetes to adhere to their medication?* | 1. Do nothing 2. Brochure/leaflet ________ 3. Consultation ________ 4. Patient group discussion ________ 5. Medication review ________ 6. Phone call refill reminder ________ 7. Others _______________ |
| *Q14. Do you have any experience to help patients with diabetes who have medication non-adherence behavior?* | 1. Yes **(please go to Q15)** 2. No |
| *Q15. For* ***patients with non-medication adherence behavior,*** *what kind of help/services have you provided?* | 1. Do nothing 2. Brochure/leaflet 3. Consultation 4. Patient group discussion 5. Medication review 6. Phone call refill reminder 7. Others _______________ |

**Section B. Pharmacist' preferences toward the type of pharmacist services**

Below are some types of services that might be offered by you as a pharmacist to help your patient taking their diabetes medication consistently as instructed or indicated in the prescription:

- **The pharmacist provides information** regarding the risk factor of getting diabetes complications, the intended blood glucose level and other measurements that may influence the development of complications (for instance, blood pressure and low-density lipoprotein/LDL) **through**:
  1. ***Brochures/leaflet***
  2. ***Face to face individual consultation***
  3. ***Patient group discussion*:** a scheduled meeting with other patients with diabetes to discuss and share information organized by the pharmacist
- ***Medication review***: pharmacist will review the patient's medication each time patients with diabetes have their medication refilled to prevent and manage any medication-related problems
- ***Phone call refill reminder***: pharmacist will remind the patient to visit the physician and refill their medication by calling the patient two or three days before their actual schedule

Please rank the five pharmacist services by placing them in the order of your preference (1= the most preferred pharmacist service; and 5= the least preferred pharmacist service). Please only use **ONE number for ONE service**. Please also identify the type of pharmacist services that you have already given to your patient until now by filing in mark () in the box.

| **Pharmacist services** | **Fill in the rank from 1-5.** | **Please** mark (V)  **for the type of services that you have already provided to the patients with diabetes until now** |
| --- | --- | --- |
| Brochure/leaflet |  |  |
| Face to face individual consultation with the pharmacist |  |  |
| Patient group discussion |  |  |
| Medication review |  |  |
| Phone call refill reminder |  |  |
