# Supplementary material for: Pharmacists’ and patients’ perceptions about the importance of pharmacist services types to improve medication adherence among patients with diabetes in Indonesia
Source: BMC Health Serv Res. 2021 Nov 13;21:1227. doi: 10.1186/s12913-021-07242-1 (PMC8590236; doi:10.1186/s12913-021-07242-1)
Supplement: Supplementary file 2 — Additional file 2. [file 12913_2021_7242_MOESM2_ESM.docx]

**Additional file 2. Social demographic and characteristics of the patients involved in the study**

|  |  | **Community health centers** | |  | **Hospitals** |  |
| --- | --- | --- | --- | --- | --- | --- |
| **Characteristics variables** | **Value range** | **Frequency** | - **Median** - **Mean ± St. Dev** | **Frequency** | - **Median** - **Mean ± St. Dev** | **Comparative test** |
| **Age** | 26 – 84 years (Community health center)  35 – 81 years (Hospital) | - | - 59.00 - 58.80 ± 9.517 | - | - 61.00 - 60.85 ± 8.941 | - T-test - Diff: 2.052 - Confidence interval: 0.923 (lower) - 3.182 (upper) |
| **Sex** | Male = 0  Female = 1 | 120 (26.30%)  337 (73.70%) | 1.00  0.74 ± 0.441 | 154 (26.60%)  425 (73.40%) | 1.00  0.73 ± 0.442 | - Two-sample Wilcoxon rank-sum (Mann-Whitney) test - Z = -0.123 - Prob > \|z\| = 0.902 |
| **Comorbidities** | No comorbidity = 0  With comorbidity = 1 | 148 (32.39%)  309 (67.61%) | 1.00  0.68 ± 0.468 | 100 (17.27%)  479 (82.73%) | 1.00  0.82 ± 0.378 | - Two-sample Wilcoxon rank-sum (Mann-Whitney) test - z = 5.658 - Prob > \|z\| = 0.000 |
| **Type of comorbidities** | No other disease  Hypertension  Heart disease  Dyslipidemia  Renal problem  Eyesight disorder or blindness  Stroke  Diabetic foot ulcer  Others | 148 (32.40%)  156 (34.10%)  21 (4.60%)  129 (28.20%)  2 (0.40%)  1 (0.20%)  -  2 (0.40%)  59 (15.8%) |  | 100 (17.30%)  268 (46.30%)  138 (23.80%)  166 (28.70%)  15 (2.60%)  12 (2.10%  21 (3.60%)  25 (4.30%)  162 (28.00%) |  |  |
| **Marital status** | Single/widowed/divorced = 0  Married/living together = 1 | 132 (28.90%)  325 (71.10%) | 1.00  0.71 ± 0.454 | 172 (29.70%)  407 (70.30%) | 1.00  0.70 ± 0.457 | - Two-sample Wilcoxon rank-sum (Mann-Whitney) test - z = -0.288 - Prob > \|z\| = 0.773 |
| **Educational background 1** | Have educational background = 0  No educational backgrounds = 1 | 425 (93.00%)  32 (7.00%) | 0.00  0.07 ± 0.255 | 560 (96.70%)  19 (3.30%) | 0  0.03 ± 0.178 | - Two-sample Wilcoxon rank-sum (Mann-Whitney) test - z = -2.747 - Prob > \|z\| = 0.006 |
| **Educational background 2** | Primary education = 1  Other educational backgrounds = 0 | 185 (40.50%)  272 (59.50%) | 0  0.40 ± 0.491 | 188 (32.50%)  391 (67.50%) | 0  0.32 ± 0.469 | - Two-sample Wilcoxon rank-sum (Mann-Whitney) test - z = -2.666 - Prob > \|z\| = 0.0077 |
| **Educational background 3** | Secondary education = 1  Other educational backgrounds = 0 | 200 (43.80%)  257 (56.20%) | 0  0.44 ± 0.497 | 292 (50.40%)  287 (49.60%) | 1  0.50 ± 0.500 | - Two-sample Wilcoxon rank-sum (Mann-Whitney) test - z = 2.133 - Prob > \|z\| = 0.0329 |
| **Work status** | Do not work/retired = 0  Work = 1 | 316 (69.10%)  141 (30.90%) | 0.00  0.31 ± 0.462 | 474 (81.90%)  105 (18.10%) | 0.00  0.18 ± 0.386 | - Two-sample Wilcoxon rank-sum (Mann-Whitney) test - z = -4.775 - Prob > \|z\| = 0.0000 |
| **Total monthly income** | < 96 USD (1.400.000 IDR)  Rp. 96 USD (1.400.000 IDR) - 138 USD (2.000.000 IDR)  ≥ 138 USD (2.000.000 IDR) | 213 (46.61%)  115 (25.16%)  129 (28.23%) |  | 194 (33.51%)  202 (34.89%)  181 (31.26%) |  | - Kruskal-Wallis equality-of-populations rank test - Chi-square with ties: 99.115 with 2 d.f. - Probability = 0.0001 |
| **Total income coverage for the household expenses** | Yes = 0  No = 1 | 383 (83.80%)  74 (16.20%) | 1  1.12 ± 0.369 | 502 (86.70%)  77 (13.30%) | 0.00  0.13 ± 0.340 | - Two-sample Wilcoxon rank-sum (Mann-Whitney) test - z = -1.310 - Prob > \|z\| = 0.1902 |
| **The need of help by others to take medication** | No = 0  Yes = 1 | 429 (93.90%)  28 (6.10%) | 0  0.06 ± 0.240 | 473 (81.70%)  106 (18.30%) | 0.00  0.18 ± 0.387 | - Two-sample Wilcoxon rank-sum (Mann-Whitney) test - z = 5.798 - Prob > \|z\| = 0.0000 |
| **Missed to take medication in the past two months** | No = 0  Yes = 1 | 273 (59.70%)  184 (40.30%) | 0  0.40 ± 0.491 | 418 (72.20%)  161 (27.80%) | 0.00  0.28 ± 0.448 | - Two-sample Wilcoxon rank-sum (Mann-Whitney) test - z = -4.222 - Prob > \|z\| = 0.0000 |
| **Experiences of getting medication information from pharmacist** | Non – pharmacist = 0  Pharmacist = 1 | 137 (29.98%)  320 (70.02%) | 1  0.79 ± 0.459 | 298 (51.47%)  281 (48.53%) | 0.00  0.49 ± 0.500 | - Two-sample Wilcoxon rank-sum (Mann-Whitney) test - z = -6.955 - Prob > \|z\| = 0.0000 |
| **Type of pharmacist service experienced by the patient** | Brochure/leaflet  Face-to-face consultation  Patient group discussion  Medication review  Phone call refill reminder  None | 117 (25.60%)  236 (51.60%)  72 (15.80%)  15 (3.30%)  4 (0.90%)  122 (26.70%) |  | 120 (20.70%)  103 (17.80%)  25 (4.30%)  12 (2.10%)  70 (12.10%)  254 (43.90%) |  |  |

**IDR=Indonesian rupiah, USD=United States Dollar**
