# Supplementary material for: Pharmacists’ and patients’ perceptions about the importance of pharmacist services types to improve medication adherence among patients with diabetes in Indonesia
Source: BMC Health Serv Res. 2021 Nov 13;21:1227. doi: 10.1186/s12913-021-07242-1 (PMC8590236; doi:10.1186/s12913-021-07242-1)
Supplement: Supplementary file 3 — Additional file 3. [file 12913_2021_7242_MOESM3_ESM.docx]

**Additional file 3. Social demographic and characteristics of pharmacists involved in the study**

|  |  | **Community health centers** | | **Hospitals** | | **Total** | |
| --- | --- | --- | --- | --- | --- | --- | --- |
| **Characteristics variables** | **Value range** | **Freq** | - **Median** - **Mean ± St.Dev** | **Freq** | - **Median** - **Mean ± St.Dev** | **Freq** | - **Median** - **Mean ± St.Dev** |
| **Age** | 23 – 44 years | - | - 34.00 - 33.57 ± 4.908 | - | - 27.50 - 29.31 ± 6.154 | - | - 32.00 - 32.02 ± 5.746 |
| **Duration of work as a pharmacist** | < 7.75 years  ≥ 7.75 years | 20 (31.75%)  43 (68.25%) | - 1.00 - 0.68 ± 0.466 | 26 (72.22%)  10 (27.78%) | - 0.00 - 0.28 ± 0.449 | 46 (46.46%)  53 (53.54%) | - 1.00 - 0.54 ± 0.499 |
| **Sex** | Male = 0  Female = 1 | 10 (15.90%)  53 (84.10%) | - 1 - 0.84 ± 0.368 | 3 (8.30%)  33 (91.70%) | - 1 - 0.92 ± 0.280 | 13 (13.10%)  86 (86.90%) | - 1 - 0.87 ± 0.339 |
| **Educational background** | Non – master’s degree = 0  Master’s degree=1 | 62 (98.40%)  1 (1.60%) | - 0 - 0.02 ± 0.126 | 30 (83.30%)  6 (16.70%) | - 0 - 0.17 ± 0.378 | 92 (92.90%)  7 (7.10%) | - 0 - 0.07 ± 0.258 |
| **Experience with helping non-adherence patient with diabetes** | No = 0  Yes = 1 | 29 (46.00%)  34 (54.00%) | - 1 - 0.54 ± 0.502 | 19 (52.80%)  17 (47.20%) | - 0 - 0.47 ± 0.506 | 48 (48.50%)  51 (51.50%) | - 1 - 0.52 ± 0.502 |
| **Type of pharmacist services already provided by the pharmacist** | Brochure/leaflet  Face-to-face consultation  Patient group discussion  Medication review  Phone call refill reminder | 49 (77.78%)  59 (93.65%)  33 (52.38%)  14 (22.22%)  4 (0.06%) |  | 28 (77.78%)  36 (100.00%)  4 (11.11%)  12 (33.33%)  3 (0.08%) |  | 77 (77.78%)  95 (95.96%)  37 (37.37%)  26 (26.26%)  7 (0.07%) |  |
