# Supplementary material for: Pharmacists’ and patients’ perceptions about the importance of pharmacist services types to improve medication adherence among patients with diabetes in Indonesia
Source: BMC Health Serv Res. 2021 Nov 13;21:1227. doi: 10.1186/s12913-021-07242-1 (PMC8590236; doi:10.1186/s12913-021-07242-1)
Supplement: Supplementary file 4 — Additional file 4. [file 12913_2021_7242_MOESM4_ESM.docx]

**Additional file 4. Marginal effect analysis for patients in community health centers and hospitals**

|  | Brochure | | Consultation | | Patient group discussion | | Medication review | | Phone call refill reminder | |
| --- | --- | --- | --- | --- | --- | --- | --- | --- | --- | --- |
|  | **CHC** | **Hospital** | **CHC** | **Hospital** | **CHC** | **Hospital** | **CHC** | **Hospital** | **CHC** | **Hospital** |
| Average overall probability | 0.2023 | 0.2695 | 0.5901 | 0.4180 | 0.0912 | 0.0651 | 0.0715 | 0.1056 | 0.0441 | 0.1401 |
| Characteristics | **Marginal effect ± standard error** | | | | | | | | | |
| Age | 0.0046 ± 0.0019* | 0.0010 ± 0.0020 | -0.0055 ± 0.0029 | -0.0016 ± 0.0023 | 0.0010 ± 0.0013 | 0.0002 ± 0.0008 | -0.0001 ± 0.0010 | -0.0007 ± 0.0011 | -0.0001 ± 0.0007 | 0.0012 ± 0.0014 |
| Female | 0.0472 ± 0.0416 | 0.0222 ± 0.0387 | -0.0587 ± 0.0572 | -0.0551 ± 0.0439 | 0.0160 ± 0.0248 | 0.0264 ± 0.0150 | -0.0008 ± 0.0203 | 0.0048 ± 0.0215 | -0.0037 ± 0.0149 | 0.0015 ± 0.0274 |
| Married | 0.0017 ± 0.0382 | 0.0272 ± 0.0374 | -0.0124 ± 0.0520 | -0.0006 ± 0.0426 | 0.0081 ± 0.0225 | 0.0096 ± 0.0142 | 0.0011 ± 0.0185 | 0.0152 ± 0.0207 | 0.0016 ± 0.0139 | -0.0512 ± 0.0265 |
| No formal education | -0.1967 ± 0.0883* | 0.1793 ± 0.1000 | 0.1899 ± 0.1270 | 0.0459 ± 0.1169 | 0.0449 ± 0.0536 | 0.0481 ± 0.0379 | -0.0064 ± 0.0437 | -0.1341 ± 0.0609* | -0.0313 ± 0.0347 | -0.1395 ± 0.0788 |
| Primary education background | -0.0460 ± 0.0639 | -0.0535 ± 0.0562 | -0.1364 ± 0.0883 | 0.0237 ± 0.0640 | 0.0601 ± 0.0394 | 0.0110 ± 0.0217 | 0.0711 ± 0.0324* | -0.0023 ± 0.0312 | 0.0506 ± 0.0255* | 0.0209 ± 0.0392 |
| Secondary education background | -0.0466 ± 0.0599 | -0.0104 ± 0.0496 | -0.0557 ± 0.0830 | -0.0190 ± 0.0565 | 0.0531 ± 0.0374 | 0.0179 ± 0.0194 | 0.0322 ± 0.0302 | 0.0081 ± 0.0276 | 0.0167 ± 0.0236 | 0.0034 ± 0.0344 |
| Work | 0.0384 ± 0.0380 | 0.0121 ± 0.0436 | -0.0210 ± 0.0531 | -0.0004 ± 0.0494 | -0.0038 ± 0.0233 | 0.0003 ± 0.0166 | -0.0192 ± 0.0191 | -0.0444 ± 0.0246 | 0.0055 ± 0.0141 | 0.0321 ± 0.0312 |
| Inability to cover household expenses | -0.1290 ± 0.0452* | 0.0610 ± 0.0467 | -0.0102 ± 0.0590 | -0.0504 ± 0.0525 | -0.0057 ± 0.0256 | 0.0367 ± 0.0176* | 0.0803 ± 0.0214* | 0.0102 ± 0.0259 | 0.0639 ± 0.0170* | -0.0576 ± 0.0347 |
| Needs of help to take medication | -0.1195 ± 0.0691 | -0.0687 ± 0.0397 | 0.1033 ± 0.0922 | 0.0194 ± 0.0450 | -0.0047 ± 0.0396 | 0.0109 ± 0.0151 | 0.0092 ± 0.0315 | 0.0339 ± 0.0219 | 0.0116 ± 0.0238 | 0.0046 ± 0.0282 |
| Have experience of missing to take medication | -0.0349 ± 0.0324 | -0.0086 ± 0.0341 | 0.0592 ± 0.0443 | -0.0281 ± 0.0387 | 0.0004 ± 0.0192 | 0.0032 ± 0.0130 | -0.0174 ± 0.0159 | -0.0083 ± 0.0189 | -0.0073 ± 0.0121 | 0.0415 ± 0.0240 |
| With comorbidities | -0.0015 ± 0.0340 | -0.0102 ± 0.0412 | -0.0614 ± 0.0467 | 0.0152 ± 0.0465 | 0.0423 ± 0.0205* | -0.0311 ± 0.0157* | 0.0102 ± 0.0165 | -0.0135 ± 0.0228 | 0.0103 ± 0.0127 | 0.0395 ± 0.0299 |
| Experience getting medication information from the pharmacist | -0.3323 ± 0.0391* | -0.1683 ± 0.0313* | 0.3609 ± 0.0481* | 0.0918 ± 0.0355* | -0.0041 ± 0.0218 | -0.0107 ± 0.0120 | -0.0589 ± 0.0184* | 0.0151 ± 0.0171 | 0.0343 ± 0.0153* | 0.0722 ± 0.0220* |
| Total monthly income ≥ 96 USD (1.400.000 IDR) | -0.1402 ± 0.0354* |  | 0.2048 ± 0.0475* |  | -0.0086 ± 0.0208 |  | -0.0420 ± 0.0172* |  | -0.0138 ± 0.0130 |  |
| Total monthly income ≥ 138 USD (2.000.000 IDR) |  | 0.0439 ± 0.0387 |  | -0.0521 ± 0.0439 |  | 0.0020 ± 0.0149 |  | -0.0395 ± 0.0217 |  | 0.0452 ± 0.0272 |

*p<0.05; CHC=community health centers, IDR=Indonesia Rupiah, USD=US Dollar
