# Supplementary material for: Pharmacists’ and patients’ perceptions about the importance of pharmacist services types to improve medication adherence among patients with diabetes in Indonesia
Source: BMC Health Serv Res. 2021 Nov 13;21:1227. doi: 10.1186/s12913-021-07242-1 (PMC8590236; doi:10.1186/s12913-021-07242-1)
Supplement: Supplementary file 5 — Additional file 5. [file 12913_2021_7242_MOESM5_ESM.docx]

**Additional file 5. Marginal effect analysis for pharmacists in community health centers and hospitals**

|  | Brochure | Consultation | Patient group discussion | Medication review | Phone call refill reminder |
| --- | --- | --- | --- | --- | --- |
|  | 0.1452 | 0.7909 | 0.0267 | 0.0105 | 0.0250 |
| **Characteristics** | **Marginal effect ± standard error** | | | | |
| **Age** | 0.0081 ± 0.0087 | -0.0059 ± 0.0119 | -0.0001 ± 0.0027 | -0.0012 ± 0.0014 | -0.0010 ± 0.0025 |
| **Female** | -0.0596 ± 0.0783 | 0.0860 ± 0.1042 | -0.0451 ± 0.0283 | -0.0026 ± 0.0115 | 0.0213 ± 0.0251 |
| **Master degree** | -0.0284 ± 0.1226 | 0.1299 ± 0.1914 | -0.0395 ± 0.0468 | -0.0195 ± 0.0223 | -0.0417 ± 0.0397 |
| **Have experience helping non-adherence patient** | -0.1145 ± 0.0596 | 0.1608 ± 0.0819 | -0.0073 ± 0.0189 | -0.0188 ± 0.0126 | -0.0199 ± 0.0177 |
| **Work duration ≥ 7.75 years** | -0.0103 ± 0.0961 | 0.0064 ± 0.1322 | 0.0210 ± 0.0316 | 0.0107 ± 0.0147 | -0.0269 ± 0.0296 |
